# Molecular autopsy and family screening in a young case of sudden cardiac death reveals an unusually severe case of *FHL1* related hypertrophic cardiomyopathy

**DOI:** 10.1002/mgg3.841

**Published:** 2019-07-10

**Authors:** Anna Gaertner‐Rommel, Jens Tiesmeier, Thomas Jakob, Bernd Strickmann, Gunter Veit, Bernd Bachmann‐Mennenga, Lech Paluszkiewicz, Karin Klingel, Uwe Schulz, Kai T. Laser, Bernd Karger, Heidi Pfeiffer, Hendrik Milting

**Affiliations:** ^1^ Klinikum der Ruhr‐Universität Bochum, Klinik für Thorax‐ und Kardiovaskularchirurgie und Erich und Hanna Klessmann‐Institut für Kardiovaskuläre Forschung und Entwicklung Herz‐ und Diabeteszentrum NRW Bad Oeynhausen Germany; ^2^ Mühlenkreiskliniken, Krankenhaus Lübbecke‐Rahden, Institut für Anästhesiologie, Intensiv‐ und Notfallmedizin, Medizin Campus OWL Ruhr‐Universität Bochum Bochum Germany; ^3^ Klinikum Herford, Universitätsklinik für Anästhesiologie, Medizin Campus OWL Ruhr‐Universität Bochum Herford Germany; ^4^ Klinikum Bielefeld‐Mitte Bielefeld Germany; ^5^ Mühlenkreiskliniken, Johannes Wesling Klinikum, Universitätsinstitut für Anästhesiologie, Intensiv‐ und Notfallmedizin, Medizin Campus OWL Ruhr‐Universität Bochum Minden Germany; ^6^ Kardiopathologie Universitätsklinikum Tübingen, Institut für Pathologie und Neuropathologie Tubingen Germany; ^7^ Zentrum für angeborene Herzfehler Herz‐ und Diabeteszentrum NRW, Klinikum der Ruhr‐Universität Bochum Bad Oeynhausen Germany; ^8^ Universitätsklinikum Münster, Institut für Rechtsmedizin Münster Germany

**Keywords:** cardiomyopathy, hypertrophic cardiomyopathy, molecular autopsy, nonsense‐mediated decay, sudden cardiac death

## Abstract

**Background:**

Hypertrophic cardiomyopathy (HCM) is a genetic cardiomyopathy with a prevalence of about 1:200. It is characterized by left ventricular hypertrophy, diastolic dysfunction and interstitial fibrosis; HCM might lead to sudden cardiac death (SCD) especially in the young.

Due to low autopsy frequencies of sudden unexplained deaths (SUD) the true prevalence of SCD and especially of HCM among SUD remains unclear. Even in cases of proven SCD genetic testing is not a routine procedure precluding appropriate risk stratification and counseling of relatives.

**Methods:**

Here we report a case of SCD in a 19‐year‐old investigated by combined forensic and molecular autopsy.

**Results:**

During autopsy of the index‐patient HCM was detected. As no other possible cause of death could be uncovered by forensic autopsy the event was classified as SCD. Molecular autopsy identified two (probably) pathogenic genetic variants in *FHL1* and *MYBPC3*. The *MYBPC3* variant had an incomplete penetrance. The *FHL1* variant was a de novo mutation. We detected reduced *FHL1* mRNA levels and no FHL1 protein in muscle samples suggesting nonsense‐mediated mRNA decay and/or degradation of the truncated protein in the SCD victim revealing a plausible disease mechanism.

**Conclusion:**

The identification of the genetic cause of the SCD contributed to the rational counseling of the relatives and risk assessment within the family. Furthermore our study revealed evidences for the pathomechanism of *FHL1* mutations.

## 1 INTRODUCTION

Sudden cardiac death (SCD) refers to an unexpected death from cardiac failure in an individual with or without preexisting heart disease within 1 hr after the onset of symptoms (Priori et al., 2015). The incidence rate of SCD ranges between 50 and 100 per 100,000 in the general population of industrial countries (Deo & Albert, 2012; Risgaard et al., 2014). In younger SCD victims (<35 years) cardiomyopathies and arrhythmogenic abnormalities predominate with incidence rates of 0.6–6.2/100,000 patients/year (Kaltman et al., 2011; Winkel et al., 2011). SCD in the young (<35 years) is estimated to have a significant genetic component (Ranthe et al., 2013).

A recently published consensus document highlights the importance of genetic screening for the identification of individuals at risk for SCD (Wellens et al., 2014). Additionally, the current guidelines of the *European Resuscitation Council* 2015 on postresuscitation care recommend screening for genetic variants only in selected cases and with survived SCD (Nolan et al., 2015). The *post mortem* protocol recommended by the *AHA/ACC/HRS guideline* (Al‐Khatib et al., 2018) underlines the relevance of genetic testing for family risk profiling not only in the surviving index patient but also for the identification of possible disease‐causing mutation carriers. To enable later genetic testing standards for handling samples are needed.

Hypertrophic cardiomyopathy (HCM) is a common inherited heart disease characterized by left ventricular (LV) hypertrophy, diastolic dysfunction, and interstitial fibrosis (Elliott et al., 2008; Gersh et al., 2011; Qintar et al., 2012). As the electrical activity of the heart may also be affected by HCM it might lead to SCD (Wexler, Elton, Pleister, & Feldman, 2009). With a prevalence of about 1 in 200 HCM is one of the most commonly inherited cardiovascular diseases (Semsarian, Ingles, Maron, & Maron, 2015) and it is thought that a significant number of HCM‐cases remains undiagnosed (Maron, Peterson, Maron, & Peterson, 1994). Frequently, SCD is the first manifestation of HCM (Hudson et al., 2019). HCM is a genetic disease, mainly transmitted as an autosomal dominant trait and is caused by mutations in more than 30 genes encoding—among other proteins—components of the sarcomere (Stenson et al., 2017). Due to notoriously low autopsy frequencies of sudden unexplained deaths (SUD) even in industrial countries the true prevalence of HCM among SUDs remains unclear. Moreover, even in cases of proven SCD genetic testing is not a routine procedure for several reasons precluding appropriate risk stratification and counseling of the relatives (Nolan et al., 2015).

Here we report an unusual case of HCM identified by combined forensic and molecular autopsy. We reveal evidences for the pathomechanism and show the impact of molecular autopsy and family screening for risk assessment within the affected family.

## 2 MATERIAL AND METHODS

### 2.1 Ethical compliance

The study conforms to the principles outlined in the Declaration of Helsinki (World Medical Association, 2013). The ethics committees of the Ruhr‐University Bochum and the Ärztekammer Westfalen‐Lippe in Münster approved the study (registry Nos. 2017‐232 or 2017‐514‐b‐8, respectively).

### 2.2 Patients and biomaterial

Blood samples for molecular genetics were collected at the day of death of the index patient (III‐9) by the emergency medical service which allowed *post mortem* molecular autopsy. Tissue samples from the left ventricle and skeletal (*musculus rectus femoris*) muscles were obtained from the proband's body during forensic autopsy. The samples were immediately snap‐frozen in liquid nitrogen after removal from the body and stored at −80°C. Samples from DCM‐patients obtained during heart transplantation were used as controls.

First‐degree relatives were examined by a cardiologist. Written informed consent for sample collection was obtained from the relatives immediately after the death of the index patient. Molecular genetics were performed after counseling the family and obtaining a second written informed consent.

### 2.3 Clinical examination of the family

The family members (II‐5, II‐6, III‐6, III‐7, IV‐1 and IV‐2) were cardiologically examined. Electrocardiogram, transthoracic echocardiography and 24 hr electrocardiography monitoring were performed in each patient. The dimensions of the left ventricle, LV ejection fraction, thickness of the interventricular septum and posterior wall as well as left ventricular mass (LVmass) and mass index (LVmass index) were obtained according to guidelines (Lang et al., 2015). The LVmass and LVmass index were calculated using the cube formula (Devereux & Reichek, 1977).

### 2.4 Genetics

Blood samples for next generation sequencing (NGS) were collected from the body about 12 hr post mortem. DNA was isolated from white blood cells using standard techniques (High Pure PCR Template Preparation Kit^®^, Roche Diagnostics GmbH, Mannheim, Germany) and prepared for cardiac gene enrichment resequencing on a MiSeq^®^ NGS system according to manufacturer's instructions (TruSight^™^ Rapid Capture Sample Preparation Kit). The index patient was screened for variants in 174 genes associated with inherited cardiac conditions using the TruSight^™^ Cardio gene panel. For variant annotation the software VariantStudio^™^ v3.0 (Illumina, San Diego, USA) was used. Family members were checked for the variants found in the index patient using Sanger Sequencing (BigDye^®^ Terminator v1.1 Cycle Sequencing Kit, ABI PRISM^®^ 3100 genetic analyzer, Applied Biosystems, Foster City, CA, USA). The variants were classified according to ACMG guidelines (Richards et al., 2015).

### 2.5 Isolation of total RNA

Total RNA was isolated from the LV myocardium or skeletal muscle using commercial kits (RNeasy, Qiagen, Hilden, Germany) as previously reported (Milting et al., 2006). For RNA isolation about 30 mg of tissue was used. Purified total RNA was quantified photometrically at 260 nm and RNA‐purity and ‐integrity were assessed using agarose‐gel electrophoresis.

### 2.6 Quantitative real‐time PCR

Reverse transcription of RNA was performed using 250 ng of total RNA and 50 units of the enzyme Superscript II (Invitrogen, Netherlands) after random priming with hexamers. Quantification of the mRNA of *FHL1* was done with 2 µl of the reverse transcription reaction as template. Real‐time PCR data were analyzed using glycerinaldehyde‐3‐phosphate‐dehydrogenase, hypoxanthine phosphoribosyltransferase‐1 and beta‐2‐microglobuline as housekeeping genes on a StepOnePlus^™^ real‐time PCR system (ThermoFisher Scientific, Waltham, Massachusetts, USA) in duplicates, respectively. Primer sequences are available from the authors upon request. The conditions for the PCR reaction were: 95°C, 10 min for initial denaturation, 40 cycles 60°C, 1 min/95°C, 15 s using Maxima Probe/ROX qPCR MasterMix (ThermoFisher Scientific). Data analysis was performed according to the MIQE guidelines (Bustin et al., 2009; Vandesompele et al., 2002). The relative quantity values were calculated using the ΔΔC_T_‐method with the geometric mean of the C_T_‐values of all three endogenous controls as reference.

### 2.7 Protein extraction, analysis and immunohistochemistry

Proteins were extracted from human myocardial or skeletal muscle tissue using RIPA‐buffer (150 mM NaCl, 1 mM EDTA, 50 mM Tris‐HCl, 1% [v/v] Nonidet^™^ P40 Substitute [Merck], 0.25% [w/v] Sodium deoxycholate, 1 mM NaF, 1 mM Na_3_VO_4_, proteinase inhibitor P2714 [Sigma‐Aldrich], pH 7.4). 25–30 mg of tissue was mixed with RIPA‐buffer (10 µl buffer/1 mg tissue) and homogenized for 40 s with Ultra‐Turrax^®^. Samples were incubated for 2 hr on ice under constant agitation. After 10 min centrifugation at 21,000 *g* and 4°C the supernatant was removed. The supernatant and pellet were stored at −80°C for further analyses using SDS‐PAGE (Mini‐Protean^®^ TGX^TM^ Precast Gel 4%–20%, Bio‐Rad, Hercules, CA, USA) and subsequent Coomassie‐R‐250 staining or Western Blot, respectively. For Western Blot a polyclonal anti‐FHL1 antibody from rabbit (HPA001040, Sigma Life Science) was used as primary antibody. As secondary antibody Rabbit IgG HRP Linked Whole Ab (from Donkey; Merck, Darmstadt, Germany) was used.

Immunohistochemical analysis was performed using rabbit anti‐FHL1 antibody (1:200) on an automated immunostainer following the manufacturer's protocol (Benchmark; Ventana Medical Systems, Tucson, AZ, USA) using the ultraView detection system (Ventana) and diaminobenzidine as a substrate. Interstitial fibrosis of myocardial ventricular tissue was determined using Masson Trichrome staining.

## 3 RESULTS

### 3.1 Forensic autopsy reveals HCM in a young patient with SCD

**Figure 1:**
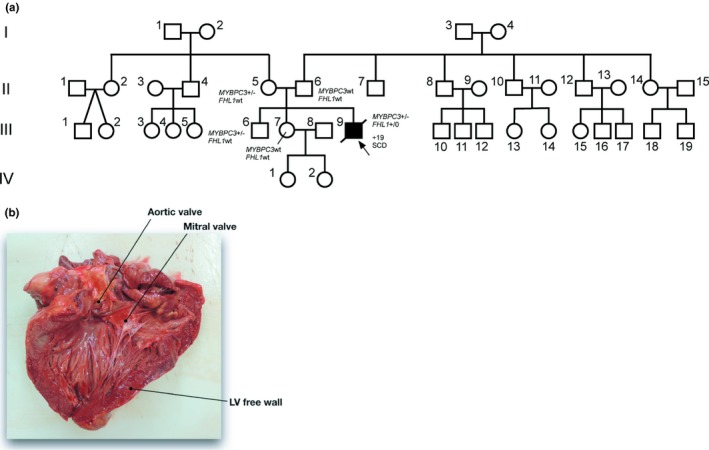
(a) Pedigree of the index patient, who died at the age of 19 years by sudden cardiac death. Relatives of the patient were examined by a cardiologist. Patient III‐9 is the only patient with a documented cardiac disease (filled symbol). (b) Explanted heart from patient III‐9 revealing a hypertrophic cardiomyopathy phenotype. The left ventricle was opened during autopsy (for data on the explanted heart s. text)

**Figure 2:**
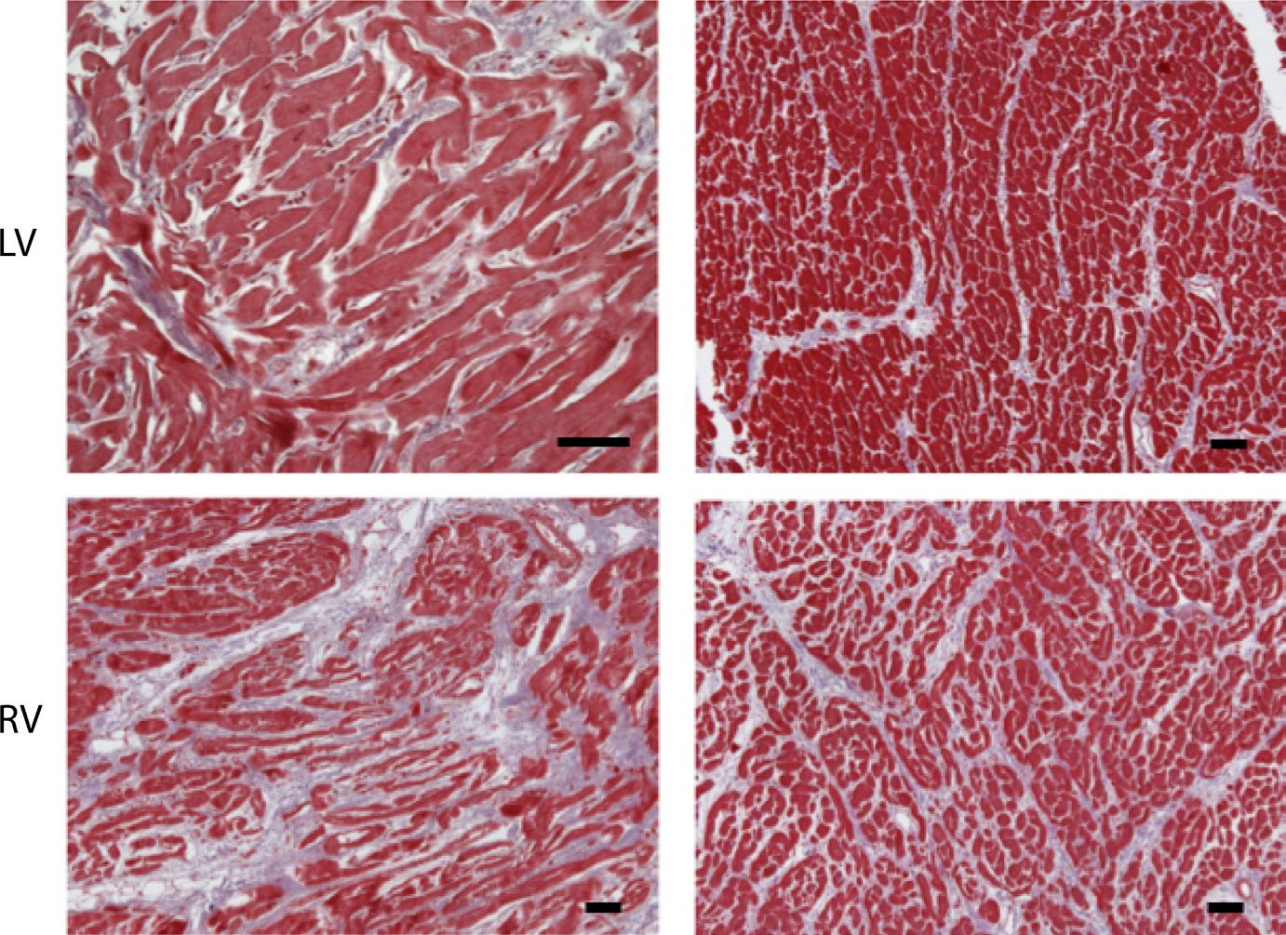
Histology from the right (RV) and left ventricle (LV) revealed hypertrophic myocytes with focal myofiber disarray and a severe diffuse interstitial fibrosis (Trichrome staining) especially in the RV. There was no significant inflammation in immunohistochemical stainings for T cells and macrophages and no evidence for an infection with cardiotropic viruses as determined using RT‐PCR (data not shown). Bars represent 100 µm

The index patient (III‐9, aged 19 years, 98 kg, 184 cm, body mass index (BMI) = 29, no known cardiac diseases; Figure 1a) died suddenly at school. A bystander cardiopulmonary resuscitation (CPR) was started immediately. The emergency medical service arrived 12 min later. The first monitored rhythm was nonshockable (asystole). Despite an Advanced Life Support according to the European Resuscitation Council Guidelines 2015 no return of spontaneous circulation could be achieved (Soara et al., 2015). Suspecting a pulmonary embolism, a fibrinolytic drug was given. After a total of 75 min the CPR attempt was terminated on scene. Histological and immunohistological analysis after forensic autopsy confirmed a primary HCM with extensive interstitial fibrosis (Figures 1b and 2). The heart weight was 638 g, wall thicknesses left (LV) 2 cm and right ventricle (RV) 0.9 cm.

### 3.2 Molecular genetics reveal pathogenic mutations in the SCD patient

Next Generation Sequencing of 174 genes associated with cardiac diseases revealed seven variants in the index patient. Two of them in *FHL1* and *MYBPC3* were classified as pathogenic or likely pathogenic according to ACMG criteria (class 4–5), respectively. Five variants in *VCL*, *CACNA1C* and *TTN* were classified as variants of unknown significance (VUS; ACMG class 3). For OMIM accession numbers and GenBank reference sequences and version numbers see Table 1. Using Sanger sequencing the patient's first‐degree relatives were tested for these seven variants (Table 1; Figure 1a).

**Table 1:** Results of panel genotyping and ACMG classification in the index patient and first‐degree relatives

gene	reference sequence	OMIM accession number	variant	ACMG‐evidence class	III‐9 (Index)	II‐5 (Mother)	II‐6 (Father)	III‐7 (Sister)	III‐6 (Brother)
*FHL1*	NM_001159702.2	300163	**p.Cys89Ter** (c.267C>A)	**5**	**+/0**	**WT**	**WT**	**WT**	**WT**
*MYBPC3*	NM_000256.3	600958	**p.Gly148Arg** (c.442G>A), rs397516050	**4**	**±**	**±**	**WT**	**WT**	**±**
*CACNA1C*	NM_199460.2	114205	**p.Ala68Thr** (c.202G>A), rs752000790	**3**	**±**	**±**	**WT**	**±**	**WT**
*VCL*	NM_014000.2	193065	**p.Pro942Ser** (c.2824C>T)	**3**	**±**	**WT**	**±**	**WT**	**WT**
*TTN*	NM_001267550.1	188840	**p.Ile35908Thr** (c.107723T>C), rs769141222	**3**	**±**	**WT**	**±**	**WT**	**WT**
*TTN*	NM_001267550.1	188840	**p.Lys33843Asn** (c.101529G>C), rs377406091	**3**	**±**	**±**	**WT**	**±**	**±**
*TTN*	NM_001267550.1	188840	**p.Glu34041Lys** (c.102121G>A), rs377600383	**3**	**±**	**±**	**WT**	**±**	**±**

Abbreviations: ACMG, American college of medical genetics (5 = pathogenic, 4 = probably pathogenic, 3 = variant of unknown significance); OMIM, Online Mendelian Inheritance in Man; WT, wildtype; ±, heterozygous, +/0 = hemizygous.

For the variants the putative amino acid exchange is shown in bold.

### 3.3 A de novo mutation in *FHL1* leads to a complete absence of FHL1 protein

The *FHL1* variant c.267C>A identified in the patient converts codon 89 to a premature stop codon (p.Cys89Ter). If the truncated protein would be expressed it would end at the end of LIM 1 domain. The variant is not listed in databases of genetic variations (Abecasis et al., 2012; Fu et al., 2013; Lek et al., 2016) or in the *Human Gene Mutation Database* (Stenson et al., 2017). According to the ACMG guidelines this variant was first classified as *likely pathogenic* (class 4) since mutations in *FHL1* resulting in a premature stop codon were previously reported to be associated with HCM (Friedrich et al., 2012; Gossios, Lopes, & Elliott, 2013). As *FHL1* is an X‐chromosomal gene the male patient carried only the affected *FHL1* allele.

**Figure 3:**
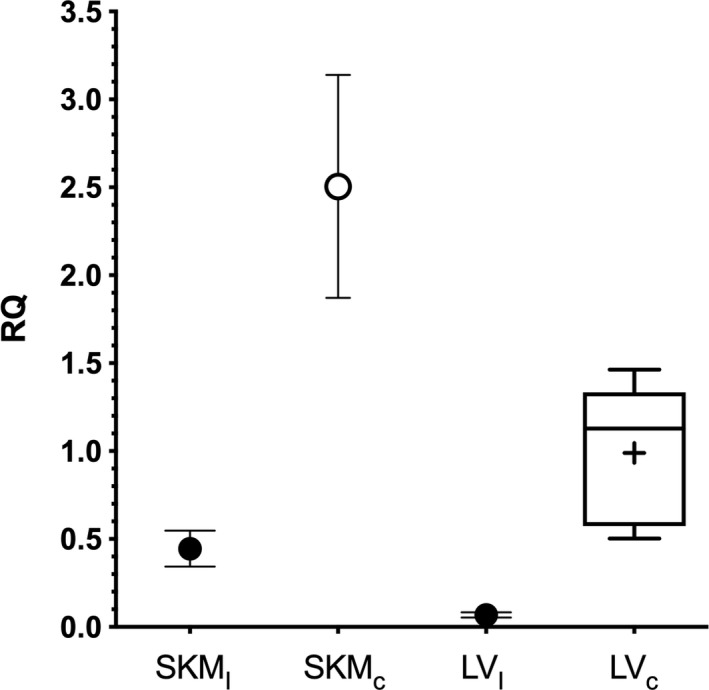
Quantitative RT‐PCR of *FHL1* mRNA expression in the index patient (III‐9) and control tissues. Samples of the skeletal muscle of the index patient (SKM_I_; 0.45 ± 0.1) and from a control individual (SKM_c_; *N* = 1; 2.51 ± 0.63) were measured as duplicates and given as means ± standard error of the mean (*SEM*). Left ventricular myocardial samples of controls without *FHL1* mutations (LV_c_; *N* = 5; box and whiskers plot: mean and median given as “+” or line, respectively; whiskers extend from 1–99 percentile) and the index patient (LV_I_; 0.07 ± 0.01). RQ, relative quantity

**Figure 4:**
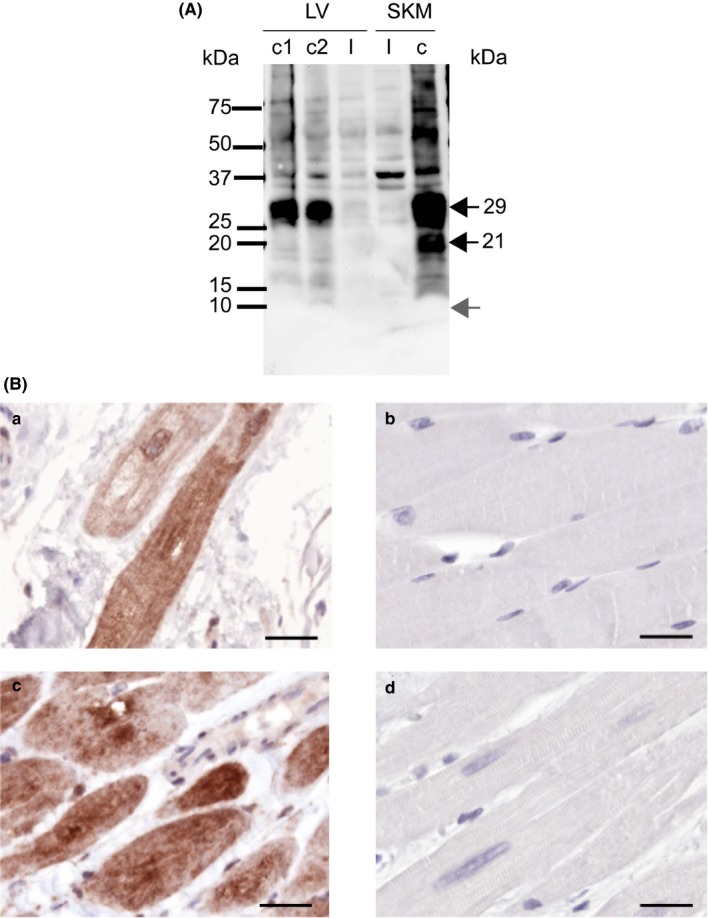
(A) Immunoblotting using an anti‐FHL1 antibody for labeling of muscle tissue extracts. The major isoforms are marked by black arrows and the apparent molecular masses are given. In preparations of the skeletal muscle (SKM) or the left ventricular myocardium (LV) of the index patient (I) FHL1 was not detectable. The molecular mass of the putative truncated peptide, as predicted from sequencing results (9.7 kDa), is marked by a gray arrow. Protein extracts of controls (C) for SKM or LV were used as a reference. (B) Immunohistochemistry of FHL1 in SKM (b) or LV (a, c, d), respectively, using diaminobenzidine as a substrate for HRP staining. Of note, FHL1‐staining was not detectable in the index patient (III‐9; b + d) but in the control tissue (a + c). Bar = 20 µm

Remarkably, genotyping of the family revealed that the *FHL1* variant was identified in the index patient (III‐9) only and is presumably a de novo mutation since a rare but possible mosaic inheritance was not further investigated. qPCR analysis of the index patient`s skeletal (*M. rectus femoris*; SKM) and LV cardiac muscle revealed reduced *FHL1* mRNA levels in the patient compared to controls (Figure 3). The *FHL1* mRNA level in the LV and SKM of the patient was reduced to 7% and 18% of the means in controls, respectively. In addition, the truncated FHL1 was not detectable in skeletal muscle and LV tissue of the index patient (III‐9) by Western blotting or immunohistochemistry, respectively (Figure 4A,B). Degradation of cardiac protein in the *post mortem* samples of the index patient could be excluded (Figure S2). Due to the absence of an intact *FHL1* allele in the hemizygous patient the gene variant results in a functional knock‐out of FHL1 probably as a consequence of nonsense‐mediated decay of the FHL1 mRNA (for the characterization of the antibody s. supplements, Figure S1).

As a consequence of the genetic analysis in the family and tissue examination on FHL1 expression the *FHL1* nonsense mutation had to be reclassified as *pathogenic* (ACMG class 5).

### 3.4 The relevance of a *MYBPC3* missense variant remains unclear

**Figure 5:**
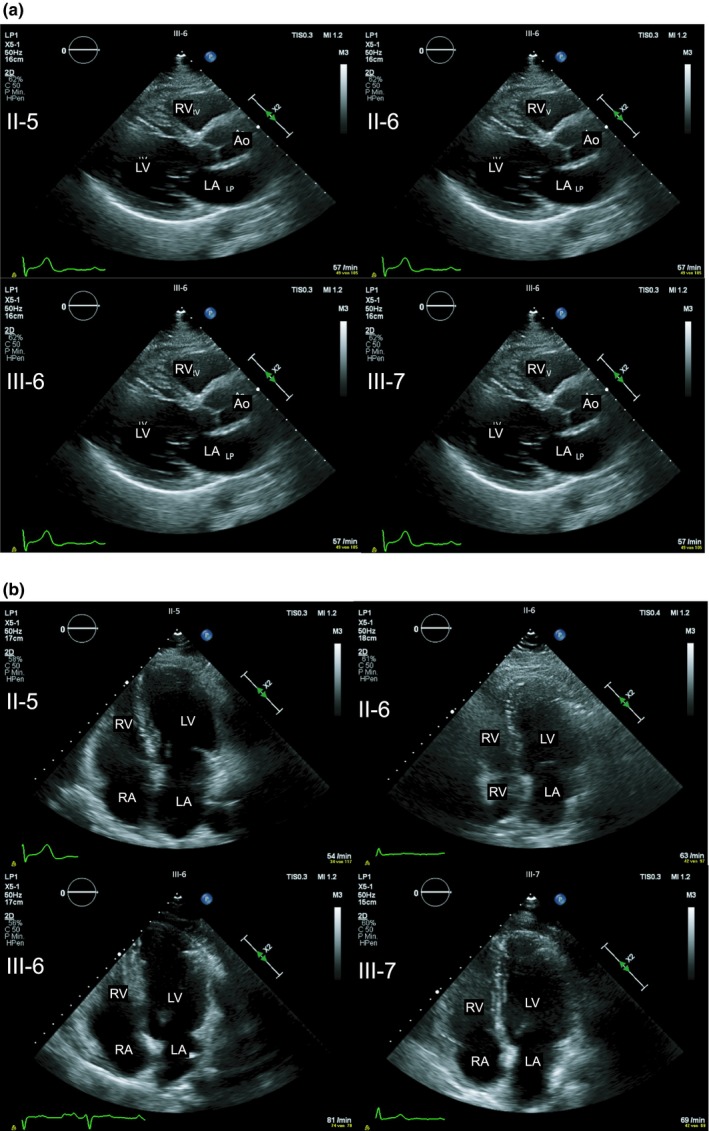
Echocardiography of family members (II‐5, II‐6, III‐6 and III‐7) reveals no further cases of hypertrophic cardiomyopathy. (a) Parasternal long axis and (b) four‐chamber view. LA = left atrium, LV = left ventricle, RA = right atrium, RV = right ventricle, Ao = aorta. For echocardiographic details s. Table 2

The *MYBPC3* missense variant was classified as *likely pathogenic* (ACMG‐class 4). This variant is listed in the ExAC browser with an allele frequency of 0.00004 (Lek et al., 2016). However, it is known from literature that this variant is associated with HCM or LVNC (Hoedemaekers et al., 2010; Wessels et al., 2015), respectively. In several cases the variant is also associated with cardiomyopathy or SCD in a compound heterozygous (Wessels et al., 2015) or digenic (Christiansen et al., 2016) genotype. Furthermore, the variant showed incomplete penetrance and variable expressivity within another family (Hoedemaekers et al., 2010). The patient`s mother (II‐5) and the patient's brother (III‐8) were also carriers of the *MYBPC3* missense variant. The variants in *VCL*, *CACNA1C* and *TTN* were not further analyzed and are reported as VUS (Table 1). Cardiological checkup of first‐degree relatives of the index patient revealed only mild myocardial hypertrophy in both parents so they have to be classified as unaffected (Figure 5a,b; Table 2).

**Table 2:** Data from transthoracic echocardiography of the relatives

	Age [y]	LVEDD [mm]	LVESD [mm]	IVS [mm]	PW [mm]	LVmass [g]	LVmass Index [g/m^2^]	LV‐EF [%]
II‐5	54	48	32	**11** (6–9)	9 (6–9)	**170** (66–150)	82 (44–88)	63%
II‐6	57	50	40	**12** (6–10)	10 (6–10)	**207** (96–200)	95 (50–102)	58%
III‐6	32	50	32	9 (6–10)	9 (6–10)	158 (96–200)	79 (50–102)	68%
III‐7	33	46	33	8 (6–9)	9 (6–9)	128 (66–150)	73 (44–88)	64%

Abbreviations: IVS, interventricular septum; LVEDD, left ventricular end diastolic diameter, (parasternal long axis); LVESD, left ventricular end systolic diameter (parasternal long axis); LVmass, left ventricular mass; LVmass index, left ventricular mass index; LV‐EF, left ventricular ejection fraction; PW, posterior wall left ventricle. Reference ranges for IVS, PW, LVmass and LVmass Index are given in brackets. Values outside of the normal range are shown in bold.

## 4 DISCUSSION

With a prevalence of 7.7/100,000 (Risgaard et al., 2014) even in patients <50 years SCD remains a challenging medical problem (Wellens et al., 2014). By definition SCD is an unexpected fatal event, with major psychosocial impact for relatives. While recent studies estimate that up to 75% of SCD cases in young patients are related to inherited heart diseases (Ferrero‐Miliani, Holst, Pehrson, Morling, & Bundgaard, 2010) *post mortem* genetic testing is currently practiced incompletely in different industrial countries. Although the costs for genetic testing drastically decreased in recent years, genetic testing remains relatively expensive and is not covered for deceased cases by health insurances in most countries. Furthermore, the frequency of autopsies for pathological examination is notoriously low and forensics is only performed if an unnatural death is suspected by prosecutors. In addition, autopsies might be done even days *post mortem* and isolated DNA of i.e. blood samples might therefore be degraded. However, the availability of high‐quality genomic DNA from autopsy is a premise for high throughput NGS and cardiogenetic analyses. We therefore launched a model study with the emergency medical service in a rural area of the German state North Rhine Westphalia to save biomaterial from young patients with need for CPR.

One of the first cases we identified in this study was an SUD‐case of a 19‐year‐old man who died at school and was transferred to forensic examination of his body. At autopsy the patient was diagnosed with a HCM and the SUD event could be classified as SCD. The LV free wall revealed macroscopically extensive interstitial fibrosis which was confirmed using histology (Figure 2). Of note, the presence of HCM in this patient was not known before autopsy since he showed no signs of cardiac disease or physical limitations before SCD. NGS‐panel DNA sequencing revealed two pathogenic mutations and five VUS.

One of the *pathogenic* mutations was identified within *MYBPC3* which is a major HCM‐associated gene. 35% of the known HCM‐associated mutations are found within *MYBPC3* (Stenson et al., 2017). Although classified as *likely pathogenic* according to ACMG criteria the relevance of the patient`s *MYBPC3*‐missense variant p.Gly148Arg (c.442G>A) for the SCD‐event is hard to predict since it was previously shown to be associated with incomplete penetrance and variable expressivity within a family (Hoedemaekers et al., 2010). The genetic variant was also previously identified in another patient with SCD. However, this patient presented a digenic mutant genotype (Christiansen et al., 2016). Genotyping within the family of the index patient revealed the presence of *MYBPC3* p.Gly148Arg also in the mother (II‐5) who showed only mild signs of LV hypertrophy at the age of 54 years (s. Table 2) and in the patient's brother (III‐6) who had no clinical phenotype of HCM at the age of 32 years. Thus, it remains unclear if *MYBPC3* p.Gly148Arg alone leads to a cardiac phenotype especially considering the pedigree which is free of any cardiac diseases (Figure 1a).

The second affected gene was *FHL1*, which is located on Xq26.3. This gene encodes the protein Four‐and‐a‐half LIM domains 1 (FHL1) which is a member of the FHL protein family and characterized by an N‐terminal half LIM domain followed by four complete LIM domains (reviewed in Cowling et al.[, 2011]). FHL1 is the main isoform in striated muscles and is supposed to contribute to sarcomere formation, assembly and biomechanical stress sensing (Chu, Ruiz‐Lozano, Zhou, Cai, & Chen, 2000; Sheikh et al., 2008). More than 25 different protein interactions have been identified for full length FHL1 and its splice variants (reviewed in (Shathasivam, Kislinger, & Gramolini, 2010)). The interactors of FHL1 include signal transducers, transcription regulators, receptors, ion‐channels and structural proteins including cardiac *myosin binding protein* (cMyBP‐C encoded by *MYBPC3*) or titin (McGrath et al., 2006; Sheikh et al., 2008). FHL1 is the only FHL member with different splice variants. Although FHL1 is classified as a LIM‐only protein, spliced variants have been identified containing additional domains resulting in differential localization patterns, protein interactions and functions.

Mutations in *FHL1* are associated with at least six different muscular dystrophies including reducing body myopathy (Jokela et al., 2017; Shalaby, Hayashi, Nonaka, Noguchi, & Nishino, 2009), muscular dystrophy (Pen et al., 2015), X‐linked myopathy (Windpassinger et al., 2008) and HCM (Binder, et al., 2012; Friedrich et al., 2012; Kubota, et al., 2019; Liang, et al., 2018; Willis, et al., 2016). In most cases the skeletal muscle disorders are coupled with cardiovascular diseases. Nonsense mutations of *FHL1* are associated with HCM and skeletal muscle weakness but also with isolated HCM (Friedrich et al., 2012).

It was observed that disease‐associated *FHL1* mutations might lead to reduced levels of FHL1 mutant proteins in cell culture, which could be rescued by proteasome inhibition (Friedrich et al., 2012). We analyzed the patient's myocardium and skeletal muscle for the presence of FHL1. The antibody, which was used for immunological detection was tested before to identify a truncated FHL1 in cell culture. However, the truncated FHL1 was not detectable in vivo by immunoblotting or immunohistochemistry, respectively. In addition, the mRNA of FHL1 was also significantly reduced when compared to controls. Therefore, we conclude that the truncated form of FHL1 is not or on an extremely low level expressed in the muscle tissue of the SCD‐victim. Since the mRNA was reduced in the muscle tissues of the patient, we conclude that nonsense mediated mRNA decay contributes to the loss of FHL1 expression in vivo.

Since the index patient carries combined pathogenic mutations in *MYBPC3* and *FHL1* it might be speculated that the joined effects of *FHL1 and MYBPC3* mutations affecting sarcomeric interaction partners might promote the development of the fatal cardiac phenotype.

Surprisingly, although the index patient showed a complete loss of FHL1 within the skeletal muscle no morphological signs of a myopathy were reported or detected during autopsy, respectively. In X‐linked reducing body myopathy caused by *FHL1* missense mutations intracytoplasmic aggregates are identified with FHL1 as the most prominent protein (Schessl et al., 2009). Our immunohistochemical analysis suggests another disease mechanism as no FHL1 was detected in the patient's muscle biopsies.

Rigid spine was identified as a common clinical feature among patients afflicted with any of the five *FHL1*‐associated X‐linked myopathies (Shalaby et al., 2008). According to the report of the index patient's mother the patient had postural defects. It is unclear if these might be interpreted as signs of a beginning myopathy. Due to the postural defects the patient started with sport activities approximately one month before his death. *FHL1*‐associated myopathies might result in muscular atrophy or hypertrophy with a pseudoathletic phenotype (Windpassinger et al., 2008). However, we have no evidence that the patients’ BMI of 29 is the result of muscular hypertrophy.

The *FHL1* variant was not found in the blood‐DNA of other family members and is probably a de novo genetic variant, explaining why none of the other family members presented an HCM phenotype. We did not find evidence for a dominant effect of the *MYBPC3* variant, since another male carrier was without a cardiological phenotype. We conclude that the combination of the two genetic variants might lead to a more severe phenotype than each variant alone stressing the impact of multigenic genotypes for the development of a fatal disease course.

The presence of at least two presumably disease‐associated genetic variants emphasizes the impact of broad genetic testing compared to a focused screening of HCM‐associated genes. It shows that further genetic screening is sensible even if a probably pathogenic mutation in one of the major HCM genes has already been found. In addition, our study points out the role of *FHL1* as a possibly underestimated HCM and SCD gene.

The present study shows the relevance of integrating emergency medicine, forensic and molecular autopsy to unravel the cause of SUD cases. Guidelines for emergency medicine, pathology and forensic medicine should be harmonized for an effective advanced care of affected families.

## CONFLICT OF INTEREST

The authors have no conflict of interest.

## References

[mgg3841-bib-0001] Abecasis, G. R. , Auton, A. , Brooks, L. D. , DePristo, M. A. , Durbin, R. M. , Handsaker, R. E. , … McVean, G. A. (2012). An integrated map of genetic variation from 1,092 human genomes. Nature, 491(7422), 56–65. 10.1038/nature11632 23128226 PMC3498066

[mgg3841-bib-0002] Al‐Khatib, S. M. , Stevenson, W. G. , Ackerman, M. J. , Bryant, W. J. , Callans, D. J. , Curtis, A. B. , … Page, R. L. (2018). 2017 AHA/ACC/HRS guideline for management of patients with ventricular arrhythmias and the prevention of sudden cardiac death: A Report of the American College of Cardiology/American Heart Association Task Force on Clinical Practice Guidelines and the Heart Rhythm Society. Heart Rhythm: The Official Journal of the Heart Rhythm Society, 15(10), e73–e189. 10.1016/j.hrthm.2017.10.036 29097319

[mgg3841-bib-0003] Binder, J. S. , Weidemann, F. , Schoser, B. , Niemann, M. , Machann, W. , Beer, M. , … Windpassinger, C. (2012). Spongious Hypertrophic Cardiomyopathy in Patients With Mutations in the Four-and-a-Half LIM Domain 1 Gene. Circulation: Cardiovascular Genetics, 5(5), 490–502. 22923418 10.1161/CIRCGENETICS.111.962332

[mgg3841-bib-0004] Bustin, S. A. , Benes, V. , Garson, J. A. , Hellemans, J. , Huggett, J. , Kubista, M. , … Wittwer, C. T. (2009). The MIQE guidelines: Minimum information for publication of quantitative real‐time PCR experiments. Clinical Chemistry, 55(4), 611–622. 10.1373/clinchem.2008.112797 19246619

[mgg3841-bib-0005] Christiansen, S. L. , Hertz, C. L. , Ferrero‐Miliani, L. , Dahl, M. , Weeke, P. E. , LuCamp, … Morling, N. (2016). Genetic investigation of 100 heart genes in sudden unexplained death victims in a forensic setting. European Journal of Human Genetics, 24(12), 1797–1802. 10.1038/ejhg.2016.118 27650965 PMC5117921

[mgg3841-bib-0006] Chu, P. H. , Ruiz‐Lozano, P. , Zhou, Q. , Cai, C. , & Chen, J. (2000). Expression patterns of FHL/SLIM family members suggest important functional roles in skeletal muscle and cardiovascular system. Mechanisms of Development, 95(1–2), 259–265. 10.1016/S0925-4773(00)00341-5 10906474

[mgg3841-bib-0007] Cowling, B. S. , Cottle, D. L. , Wilding, B. R. , D'Arcy, C. E. , Mitchell, C. A. , & McGrath, M. J. (2011). Four and a half LIM protein 1 gene mutations cause four distinct human myopathies: A comprehensive review of the clinical, histological and pathological features. Neuromuscular Disorders, 21(4), 237–251. 10.1016/j.nmd.2011.01.001 21310615

[mgg3841-bib-0008] Deo, R. , & Albert, C. M. (2012). Epidemiology and genetics of sudden cardiac death. Circulation, 125(4), 620–637. 10.1161/CIRCULATIONAHA.111.023838 22294707 PMC3399522

[mgg3841-bib-0009] Devereux, R. B. , & Reichek, N. (1977). Echocardiographic determination of left ventricular mass in man. Anatomic validation of the method. Circulation, 55(4), 613–618. 10.1161/01.CIR.55.4.613 138494

[mgg3841-bib-0010] Elliott, P. , Andersson, B. , Arbustini, E. , Bilinska, Z. , Cecchi, F. , Charron, P. , … Keren, A. (2008). Classification of the cardiomyopathies: A position statement from the European Society of Cardiology Working Group on Myocardial and Pericardial Diseases. European Heart Journal, 29(2), 270–276. 10.1093/eurheartj/ehm342 17916581

[mgg3841-bib-0011] Ferrero‐Miliani, L. , Holst, A. G. , Pehrson, S. , Morling, N. , & Bundgaard, H. (2010). Strategy for clinical evaluation and screening of sudden cardiac death relatives. Fundamental & Clinical Pharmacology, 24(5), 619–635. 10.1111/j.1472-8206.2010.00864.x 20698891

[mgg3841-bib-0012] Friedrich, F. W. , Wilding, B. R. , Reischmann, S. , Crocini, C. , Lang, P. , Charron, P. , … Carrier, L. (2012). Evidence for FHL1 as a novel disease gene for isolated hypertrophic cardiomyopathy. Human Molecular Genetics, 21(14), 3237–3254. 10.1093/hmg/dds157 22523091

[mgg3841-bib-0013] Fu, W. , O’Connor, T. D. , Jun, G. , Kang, H. M. , Abecasis, G. , Leal, S. M. , … Akey, J. M. (2013). Analysis of 6,515 exomes reveals the recent origin of most human protein‐coding variants. Nature, 493(7431), 216–220. 10.1038/nature11690 23201682 PMC3676746

[mgg3841-bib-0014] Gersh, B. J. , Maron, B. J. , Bonow, R. O. , Dearani, J. A. , Fifer, M. A. , & Link, M. S. . … Yancy, C. W. ; American College of Cardiology Foundation/American Heart Association Task Force on Practice Guidelines . (2011). 2011 ACCF/AHA Guideline for the Diagnosis and Treatment of Hypertrophic Cardiomyopathy: A report of the American College of Cardiology Foundation/American Heart Association Task Force on Practice Guidelines. Developed in collaboration with the American Association for Thoracic Surgery, American Society of Echocardiography, American Society of Nuclear Cardiology, Heart Failure Society of America, Heart Rhythm Society, Society for Cardiovascular Angiography and Interventions, and Society of Thoracic Surgeons. Journal of the American College of Cardiology, 58(25), e212–e260. 10.1016/j.jacc.2011.06.011 22075469

[mgg3841-bib-0015] Gossios, T. D. , Lopes, L. R. , & Elliott, P. M. (2013). Left ventricular hypertrophy caused by a novel nonsense mutation in FHL1. European Journal of Medical Genetics, 56(5), 251–255. 10.1016/j.ejmg.2013.03.001 23500067

[mgg3841-bib-0016] Hoedemaekers, Y. M. , Caliskan, K. , Michels, M. , Frohn‐Mulder, I. , van der Smagt, J. J. , Phefferkorn, J. E. , … Majoor‐Krakauer, D. F. (2010). The importance of genetic counseling, DNA diagnostics, and cardiologic family screening in left ventricular noncompaction cardiomyopathy. Circulation: Cardiovascular Genetics, 3(3), 232–239. 10.1161/CIRCGENETICS.109.903898 20530761

[mgg3841-bib-0017] Hudson, J. , Sturm, A. C. , Salberg, L. , Brennan, S. , Quinn, G. P. , & Vadaparampil, S. T. (2019). Disclosure of diagnosis to at‐risk relatives by individuals diagnosed with hypertrophic cardiomyopathy (HCM). Journal of Community Genetics, 10(2), 207–217. 10.1007/s12687-018-0377-1 30121752 PMC6435759

[mgg3841-bib-0018] Jokela, M. , Huovinen, S. , Palmio, J. , Saukkonen, A. M. , Penttila, S. , & Udd, B. (2017). Gluteus maximus hypertrophy: A diagnostic clue in four and a half LIM domain 1‐mutated reducing body myopathy. Neuromuscular Disorders, 27(10), 962–963. 10.1016/j.nmd.2017.06.014 28694073

[mgg3841-bib-0019] Kaltman, J. R. , Thompson, P. D. , Lantos, J. , Berul, C. I. , Botkin, J. , Cohen, J. T. , … Friedman, R. A. (2011). Screening for sudden cardiac death in the young: Report from a national heart, lung, and blood institute working group. Circulation, 123(17), 1911–1918. 10.1161/CIRCULATIONAHA.110.017228 21537007

[mgg3841-bib-0020] Kubota, A. , Juanola-Falgarona, M. , Emmanuele, V. , Sanchez-Quintero, M. J. , Kariya, S. , Sera, F. , … Hirano, M. (2019). Cardiomyopathy and altered integrin-actin signaling in Fhl1 mutant female mice. Human Molecular Genetics, 28, 209–219. 30260394 10.1093/hmg/ddy299 PMC7594173

[mgg3841-bib-0021] Lang, R. M. , Badano, L. P. , Mor‐Avi, V. , Afilalo, J. , Armstrong, A. , Ernande, L. , … Voigt, J.‐U. (2015). Recommendations for cardiac chamber quantification by echocardiography in adults: An update from the American Society of Echocardiography and the European Association of Cardiovascular Imaging. Journal of the American Society of Echocardiography, 28(1), 1–39.e14. 10.1016/j.echo.2014.10.003 25559473

[mgg3841-bib-0022] Lek, M. , Karczewski, K. J. , Minikel, E. V. , Samocha, K. E. , Banks, E. , Fennell, T. , … MacArthur, D. G. (2016). Analysis of protein‐coding genetic variation in 60,706 humans. Nature, 536(7616), 285–291. 10.1038/nature19057 27535533 PMC5018207

[mgg3841-bib-0023] Liang, Y. , Bradford, W. H. , Zhang, J. , & Sheikh, F. (2018). Four and a half LIM domain protein signaling and cardiomyopathy. Biophysical Reviews, 10(4), 1073–1085. 29926425 10.1007/s12551-018-0434-3 PMC6082310

[mgg3841-bib-0024] Maron, B. J. , Peterson, E. E. , Maron, M. S. , & Peterson, J. E. (1994). Prevalence of hypertrophic cardiomyopathy in an outpatient population referred for echocardiographic study. American Journal of Cardiology, 73(8), 577–580. 10.1016/0002-9149(94)90337-9 8147304

[mgg3841-bib-0025] McGrath, M. J. , Cottle, D. L. , Nguyen, M.‐A. , Dyson, J. M. , Coghill, I. D. , Robinson, P. A. , … Brown, S. (2006). Four and a half LIM protein 1 binds myosin‐binding protein C and regulates myosin filament formation and sarcomere assembly. Journal of Biological Chemistry, 281(11), 7666–7683. 10.1074/jbc.M512552200 16407297

[mgg3841-bib-0026] Milting, H. , Scholz, C. , Arusoglu, L. , Freitag, M. , Cebulla, R. , Jaquet, K. , … Pieske, B. (2006). Selective upregulation of beta1‐adrenergic receptors and dephosphorylation of troponin I in end‐stage heart failure patients supported by ventricular assist devices. Journal of Molecular and Cellular Cardiology, 41(3), 441–450. 10.1016/j.yjmcc.2006.04.010 16765375

[mgg3841-bib-0027] Nolan, J. P. , Soar, J. , Cariou, A. , Cronberg, T. , Moulaert, V. R. M. , Deakin, C. D. , … Sandroni, C. (2015). European Resuscitation Council and European Society of Intensive Care Medicine Guidelines for Post‐resuscitation Care 2015: Section 5 of the European Resuscitation Council Guidelines for Resuscitation 2015. Resuscitation, 95, 202–222. 10.1016/j.resuscitation.2015.07.018 26477702

[mgg3841-bib-0028] Pen, A. E. , Nyegaard, M. , Fang, M. , Jiang, H. , Christensen, R. , Mølgaard, H. , … Jensen, U. B. (2015). A novel single nucleotide splice site mutation in FHL1 confirms an Emery‐Dreifuss plus phenotype with pulmonary artery hypoplasia and facial dysmorphology. European Journal of Medical Genetics, 58(4), 222–229. 10.1016/j.ejmg.2015.02.003 25724586

[mgg3841-bib-0029] Priori, S. G. , Blomstrom‐Lundqvist, C. , Mazzanti, A. , Blom, N. , Borggrefe, M. , Camm, J. … VanVeldhuisen, D. J. ; Task Force for the Management of Patients with Ventricular Arrhythmias and the Prevention of Sudden Cardiac Death of the European Society of Cardiology (ESC) . (2015). 2015 ESC Guidelines for the management of patients with ventricular arrhythmias and the prevention of sudden cardiac death: The Task Force for the Management of Patients with Ventricular Arrhythmias and the Prevention of Sudden Cardiac Death of the European Society of Cardiology (ESC)Endorsed by: Association for European Paediatric and Congenital Cardiology (AEPC). Europace, 17(11), 1601–1687. 10.1093/europace/euv319 26318695

[mgg3841-bib-0030] Qintar, M. , Morad, A. , Alhawasli, H. , Shorbaji, K. , Firwana, B. , Essali, A. , & Kadro, W. (2012). Pacing for drug‐refractory or drug‐intolerant hypertrophic cardiomyopathy. Cochrane Database of Systematic Reviews, (5), CD008523. 10.1002/14651858.CD008523.pub2 PMC8094451 22592731

[mgg3841-bib-0031] Ranthe, M. F. , Winkel, B. G. , Andersen, E. W. , Risgaard, B. , Wohlfahrt, J. , Bundgaard, H. , … Boyd, H. A. (2013). Risk of cardiovascular disease in family members of young sudden cardiac death victims. European Heart Journal, 34(7), 503–511. 10.1093/eurheartj/ehs350 23150455

[mgg3841-bib-0032] Richards, S. , Aziz, N. , Bale, S. , Bick, D. , Das, S. , Gastier‐Foster, J. , … Rehm, H. L. (2015). Standards and guidelines for the interpretation of sequence variants: A joint consensus recommendation of the American College of Medical Genetics and Genomics and the Association for Molecular Pathology. Genetics in Medicine, 17(5), 405–424. 10.1038/gim.2015.30 25741868 PMC4544753

[mgg3841-bib-0033] Risgaard, B. , Winkel, B. G. , Jabbari, R. , Behr, E. R. , Ingemann‐Hansen, O. , Thomsen, J. L. , … Tfelt‐Hansen, J. (2014). Burden of sudden cardiac death in persons aged 1 to 49 years: Nationwide study in Denmark. Circulation: Arrhythmia and Electrophysiology, 7(2), 205–211. 10.1161/CIRCEP.113.001421 24604905

[mgg3841-bib-0034] Schessl, J. , Taratuto, A. L. , Sewry, C. , Battini, R. , Chin, S. S. , Maiti, B. , … Bönnemann, C. G. (2009). Clinical, histological and genetic characterization of reducing body myopathy caused by mutations in FHL1. Brain, 132(2), 452–464. 10.1093/brain/awn325 19181672 PMC2724920

[mgg3841-bib-0035] Semsarian, C. , Ingles, J. , Maron, M. S. , & Maron, B. J. (2015). New perspectives on the prevalence of hypertrophic cardiomyopathy. Journal of the American College of Cardiology, 65(12), 1249–1254. 10.1016/j.jacc.2015.01.019 25814232

[mgg3841-bib-0036] Shalaby, S. , Hayashi, Y. K. , Goto, K. , Ogawa, M. , Nonaka, I. , Noguchi, S. , & Nishino, I. (2008). Rigid spine syndrome caused by a novel mutation in four‐and‐a‐half LIM domain 1 gene (FHL1). Neuromuscular Disorders, 18(12), 959–961. 10.1016/j.nmd.2008.09.012 18952429

[mgg3841-bib-0037] Shalaby, S. , Hayashi, Y. K. , Nonaka, I. , Noguchi, S. , & Nishino, I. (2009). Novel FHL1 mutations in fatal and benign reducing body myopathy. Neurology, 72(4), 375–376. 10.1212/01.wnl.0000341311.84347.a0 19171836

[mgg3841-bib-0038] Shathasivam, T. , Kislinger, T. , & Gramolini, A. O. (2010). Genes, proteins and complexes: The multifaceted nature of FHL family proteins in diverse tissues. Journal of Cellular and Molecular Medicine, 14(12), 2702–2720. 10.1111/j.1582-4934.2010.01176.x 20874719 PMC3822721

[mgg3841-bib-0039] Sheikh, F. , Raskin, A. , Chu, P.‐H. , Lange, S. , Domenighetti, A. A. , Zheng, M. , … Chen, J. U. (2008). An FHL1‐containing complex within the cardiomyocyte sarcomere mediates hypertrophic biomechanical stress responses in mice. Journal of Clinical Investigation, 118(12), 3870–3880. 10.1172/JCI34472 19033658 PMC2575833

[mgg3841-bib-0040] Soara, J. , Nolanb, J. P. , Böttigerd, B. W. , Perkinse, G. D. , Lottg, C. , Carlih, P. , … Deakin, C. D. ; Adult advanced life support section Collaborators . (2015). European Resuscitation Council Guidelines for Reuscitation 2015 Section 3. Adult advanced life support. Resuscitation, 95, 100–147. 10.1016/j.resuscitation.2015.07.016 26477701

[mgg3841-bib-0041] Stenson, P. D. , Mort, M. , Ball, E. V. , Evans, K. , Hayden, M. , Heywood, S. , … Cooper, D. N. (2017). The Human Gene Mutation Database: Towards a comprehensive repository of inherited mutation data for medical research, genetic diagnosis and next‐generation sequencing studies. Human Genetics, 136(6), 665–677. 10.1007/s00439-017-1779-6 28349240 PMC5429360

[mgg3841-bib-0042] Vandesompele, J. , De Preter, K. , Pattyn, F. , Poppe, B. , Van Roy, N. , De Paepe, A. , & Speleman, F. (2002). Accurate normalization of real‐time quantitative RT‐PCR data by geometric averaging of multiple internal control genes. Genome Biology, 3(7). RESEARCH0034. 10.1186/gb-2002-3-7-research0034 PMC126239 12184808

[mgg3841-bib-0043] Wellens, H. J. J. , Schwartz, P. J. , Lindemans, F. W. , Buxton, A. E. , Goldberger, J. J. , Hohnloser, S. H. , … Wilde, A. A. (2014). Risk stratification for sudden cardiac death: Current status and challenges for the future. European Heart Journal, 35(25), 1642–1651. 10.1093/eurheartj/ehu176 24801071 PMC4076664

[mgg3841-bib-0044] Wessels, M. W. , Herkert, J. C. , Frohn‐Mulder, I. M. , Dalinghaus, M. , van den Wijngaard, A. , de Krijger, R. R. , … Dooijes, D. (2015). Compound heterozygous or homozygous truncating MYBPC3 mutations cause lethal cardiomyopathy with features of noncompaction and septal defects. European Journal of Human Genetics, 23(7), 922–928. 10.1038/ejhg.2014.211 25335496 PMC4463499

[mgg3841-bib-0045] Wexler, R. K. , Elton, T. , Pleister, A. , & Feldman, D. (2009). Cardiomyopathy: An overview. American Family Physician, 79(9), 778–784. PMC2999879 20141097

[mgg3841-bib-0046] Willis, T. A. , Wood, C. L. , Hudson, J. , Polvikoski, T. , Barresi, R. , Lochmüller, H. , … Straub, V. (2016). Muscle hypertrophy as the presenting sign in a patient with a complete deletion. Clinical Genetics, 90(2), 166–170. 27409453 10.1111/cge.12695

[mgg3841-bib-0047] Windpassinger, C. , Schoser, B. , Straub, V. , Hochmeister, S. , Noor, A. , Lohberger, B. , … Quasthoff, S. (2008). An X‐linked myopathy with postural muscle atrophy and generalized hypertrophy, termed XMPMA, is caused by mutations in FHL1. American Journal of Human Genetics, 82(1), 88–99. 10.1016/j.ajhg.2007.09.004 18179888 PMC2253986

[mgg3841-bib-0048] Winkel, B. G. , Holst, A. G. , Theilade, J. , Kristensen, I. B. , Thomsen, J. L. , Ottesen, G. L. , … Tfelt‐Hansen, J. (2011). Nationwide study of sudden cardiac death in persons aged 1–35 years. European Heart Journal, 32(8), 983–990. 10.1093/eurheartj/ehq428 21131293

[mgg3841-bib-0049] World Medical Association . (2013). World Medical Association Declaration of Helsinki: ethical principles for medical research involving human subjects. JAMA, 310(20), 2191–2194. 24141714 10.1001/jama.2013.281053

