# Supplementary material for: Molecular autopsy and family screening in a young case of sudden cardiac death reveals an unusually severe case of FHL1 related hypertrophic cardiomyopathy
Source: Mol Genet Genomic Med. 2019 Jul 10;7(8):e841. doi: 10.1002/mgg3.841 (PMC6687666; doi:10.1002/mgg3.841)
Supplement: Supplementary file 1 [file MGG3-7-e841-s001.docx]

**Supplementary information**

*Anti FHL1-antibody testing*

We tested the polyclonal antibody used for immunoblotting and immunohistochemistry of muscle tissue extracts on its specificity to detect also a truncated fragment of FHL1, which might potentially be translated in the myocardium of the index patient. As a control (**C**), untransfected HEK293 cells were analysed.

We found that the antibody is able to detect the GFP-tagged full length (**FL**; 57.4 kDa) and the truncated (**TV**; 36.3 kDa) variant of FHL1 translated from cDNA in HEK293 cultures (Fig. S1).

*Plasmid construction for antibody testing*

A clone containing the human *FHL1* open reading frame corresponding to NM_001449 with an N-Terminal N-GFPSpark^®^-Tag was obtained from Sino Biological Inc. (Beijing, China). The *FHL1*-mutant containing the premature stop codon was generated by site directed mutagenesis using the QuikChange^®^ Lightning Site-Directed Mutagenesis Kit (Agilent Technologies, Santa Clara, USA) and appropriate mutagenesis primers. All primer sequences are available on request. Plasmid identity was confirmed via Sanger Sequencing (Macrogen Europe, Amsterdam, Netherlands).

*Cell culture and transient transfection*

HEK293 cells were cultivated in DMEM (*High Glucose*, L-glutamine, HEPES, *Phenol Red*; Gibco^®^/Life Technologies) supplemented with 10% fetal calf serum (PAA, GE Healthcare, Chalfont St Giles, Great Britain), 100 u/mL penicillin (Merck) and 0.1 % (w/v) streptomycin (Merck). For all transfections Lipofectamine^TM^ 2000 (LFA; ThermoFisher Scientific) was used according to the manufacturer’s instructions. For transfection cells were plated in a 6-well plate at a density of 750,000 cells/well. After 24h transient transfections were performed with 2,500 ng plasmid DNA. Protein extraction was performed 48h post transfection.

*Protein extraction, analysis and histochemistry*

Proteins were extracted from cells using RIPA-buffer. Cells were washed with PBS, treated with a trypsin/EDTA solution (Trypsin 0.05%/EDTA 0.02% in PBS, Merck) for 5 min at 37 °C, harvested and pelleted. The cell pellet was washed with PBS and supplied with 50 µL RIPA buffer/1*10^6^ cells. Samples were incubated for 2 h on ice under constant agitation. After 10 min centrifugation at 21,000 g and 4 °C the supernatant was removed. Supernatant and pellet were stored at -80 °C for further analyses using SDS-PAGE and subsequent Coomassie-R-250 staining or Western Blot, respectively. For Western Blot a polyclonal anti GFP/eGFP antibody from rabbit (ABIN5542746, antibodies-online) and a polyclonal anti-FHL1 antibody from rabbit (HPA001040, Sigma Life Science, USA) were used as primary antibodies. As secondary antibody Rabbit IgG HRP Linked Whole Ab (from Donkey; Merck) was used.

***Fig. S1****.* *Protein extracts from HEK293 cells analysed by immunoblotting using the anti-FHL1 and anti-eGFP antibody for detection, respectively. The full length (****FL****) and the truncated variant (****TV****) of FHL1 translated from the cDNA in HEK293 are detected by the antibodies. The expected molecular masses of the tagged FHL1 are for the full-length construct (****FL****) 57.4 kDa, and for the truncated variant (****TV****) 36.3 kDa. Extracts of non-transfected HEK293 cells are given as control (****C****).*

*Muscle tissue protein extracts*

We tested the muscle tissue derived from forensic autopsy on protein degradation using polyacrylamide gel electrophoresis and Coomassie-R-250 staining. We did not find evidences that cardiac protein might be degraded *post mortem* until the autopsy (Fig. S2).

***Fig. S2****.* *Protein extracts from left ventricular cardiac (****LV****) and skeletal (****SKM****) muscles were separated by SDS PAGE and stained by Coomassie-R-250. Of note, protein extracts of the autopsy samples (****I****) reveal distinct banding patterns comparable to extracts of snap frozen tissues from heart transplantation (****c1*** *&* ***c2****) or SKM biopsies (****c****) as controls, respectively.*
